# Supplementation with a Natural Source of Amino Acids, Sil-Q1 (Silk Peptide), Enhances Natural Killer Cell Activity: A Redesigned Clinical Trial with a Reduced Supplementation Dose and Minimized Seasonal Effects in a Larger Population

**DOI:** 10.3390/nu13092930

**Published:** 2021-08-24

**Authors:** Jung Min Cho, Dokyeong Yoo, Jeong-Yong Lee, Mi-Sun Oh, Ki-Chan Ha, Hyang-Im Baek, Seung-Min Lee, Jong Ho Lee, Hye Jin Yoo

**Affiliations:** 1 National Leading Research Laboratory of Clinical Nutrigenetics/Nutrigenomics, Department of Food and Nutrition, College of Human Ecology, Yonsei University, Seoul 03722, Korea; jm092477@naver.com (J.M.C.); ehrud7982@naver.com (D.Y.); jhleeb@yonsei.ac.kr (J.H.L.); 2 WORLDWAY Co., Ltd., Sejong-si 30003, Korea; dalgoozi@hanmail.net (J.-Y.L.); ms.oh@worldway.co.kr (M.-S.O.); 3 Healthcare Claims & Management Inc., Jeonju 54810, Korea; omphalos9121@hanmail.net (K.-C.H.); hyangim100@gmail.com (H.-I.B.); 4 Brain Korea 21 PLUS Project, Department of Food and Nutrition, College of Human Ecology, Yonsei University, Seoul 03722, Korea; leeseungmin@yonsei.ac.kr; 5 Research Center for Silver Science, Institute of Symbiotic Life-TECH, Yonsei University, Seoul 03722, Korea

**Keywords:** functional food, Sil-Q1, silk peptide, immunostimulatory effect, natural killer cell, cytokines

## Abstract

The aim of this study was to re-validate the changes in natural killer (NK) cell cytotoxicity and cytokines related to T cells after Sil-Q1 (SQ; silk peptide) supplementation in a larger pool of Korean adults with minimized daily dose of SQ and controlling seasonal influence compared to the previous study. A total of 130 subjects were randomly assigned (1:1) to consume either 7.5 g of SQ or placebo for 8 weeks. NK cell cytotoxicity and cytokines were measured at T0 (baseline) and T8 (follow-up). Comparing the NK cell cytotoxicity values at T0 and T8 within each group, the cytotoxicity at all effector cell (E) to target cell (T) ratios of 10:1, 5:1, 2.5:1, and 1.25:1 was significantly increased in the SQ group at T8. Additionally, significant differences in the changed value (Δ, subtract baseline values from follow-up values) comparison between the groups at E:T = 10:1, 5:1, and 2.5:1 were found. As a secondary endpoint, the interleukin (IL)-12 level in the SQ group was significantly increased for 8 weeks, and Δ IL-12 in the SQ group was greater than in the placebo group. In conclusion, the present study showed considerable practical implications of SQ supplementation. Thus, SQ is an effective and safe functional food supplement for enhancing immune function.

## 1. Introduction

Elderly populations are vulnerable to alterations and disorders of immune function with age [1,2]. In the last decade, some studies have reported that dietary interventions can reduce the risk of deterioration of the immune system [3,4,5]. In particular, functional food plays a crucial role in physical well-being and advancing physiological functions as a concept of preventive medicine [6,7,8].

Silk is a fiber made by silkworms and has been used only as a high-quality material for clothing for a long time. The amino acids that compose silk activate cells and tissues, causing the normal growth and division of cells. Silk peptide, also known by the brand name ‘Sil-Q1 (SQ)’, is an amino acid complex produced by acid hydrolysis of the silk cocoon. It is composed of a complex of 16 amino acids (including nonessential and essential amino acids) and peptides and is rich in glycine, alanine, and serine (indicator ingredients that are approved by the Korea Ministry of Food and Drug Safety (KMFDS), No. 2012-18). Amino acid supplementation by silk peptide is reported to have a positive effect on immunity, specifically on the maturation and cytolytic activity of natural killer (NK) cells [9,10] and other anti-inflammatory mechanisms [11,12].

Nutrition and immunocompetence have a very close relationship with regulatory T cells, and T cell-mediated immunity is greatly affected by the environment, such as nutrient absorption and dietary changes [13]. NK cell cytotoxicity, which has shown the ability to produce cytokines and chemokines by T cell activation and the ability to lyse target cells [14], is a crucial component of the immune system. The immunomodulatory properties of functional foods can be confirmed by various study designs and parameters. Indeed, a rigorous randomized, double-blind, placebo-controlled trial (RCT) with an NK cell cytotoxicity efficacy test has been introduced as the most sophisticated and conclusive research method [15,16,17]. Furthermore, a confirmatory trial, which is implemented when it is necessary to provide additional or firm evidence of efficacy or safety [18], can validate reliable efficacy results.

The goal of this study was to explore the changes in NK cell cytotoxicity and cytokines related to T cells after supplementation with SQ. Our previous study showed that 8 weeks of SQ supplementation was beneficial for the enhancement of immune status, especially NK cell activation [19]. However, among the previous study’s limitations were the lack of a ban on the influenza vaccination because of subjects’ rights, safety, and welfare, and the small size of the subject group (placebo vs. test, 30 vs. 30).

Thus, we redesigned the current confirmatory clinical trial with a minimized daily dose (previous dose of 9 g to current dose of 7.5 g), larger subject groups (total 130 adults, 1:1 randomization), and reduced seasonal influence.

## 2. Methods

### 2.1. Study Design and Ethical Review

**Figure 1:**
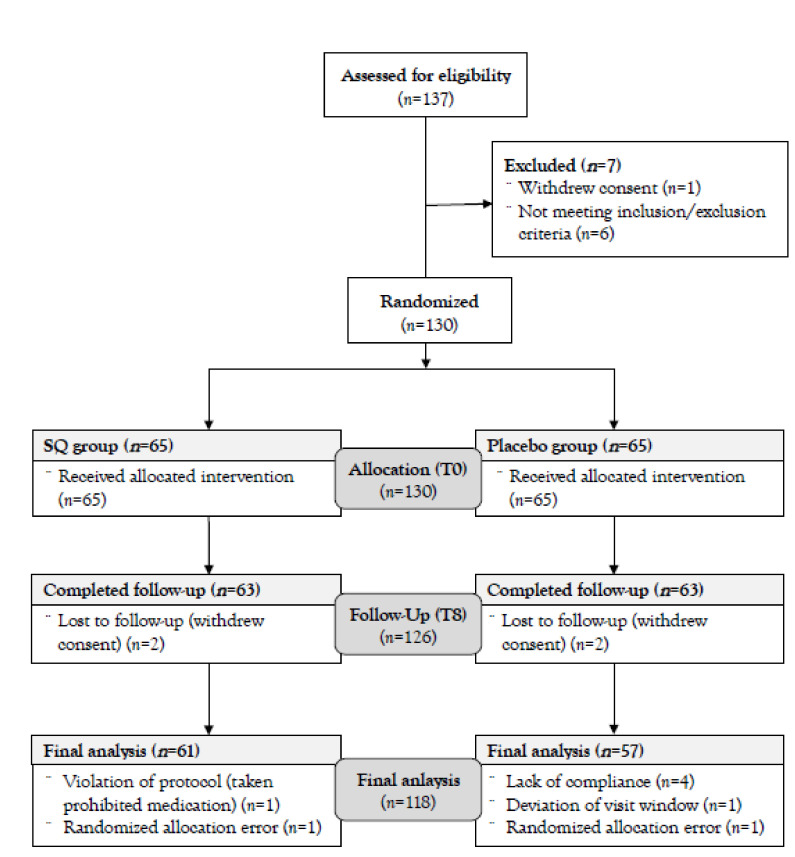
Flow diagram of the present intervention study: 137 subjects were screened before participating in the study, and a total of 130 subjects were finally enrolled. The subjects were randomly assigned to either the SQ or the placebo group at a ratio of 1:1. During the study period, 2 subjects in each group withdrew their study consent, and 2 and 6 subjects were excluded from the SQ and the placebo groups respectively, for the final analysis. Finally, a total of 118 subjects (SQ group: *n* = 61, placebo group: *n* = 57) were included in the final analysis. SQ: Sil-Q1. T0: baseline. T8: follow-up.

The study employed a single-center, randomized, placebo-controlled, parallel design for 8 weeks. The overall flow diagram of this RCT is outlined in Figure 1. First, we screened 137 subjects and enrolled 130 subjects, and then they were randomly allocated (blocked randomization) at a ratio of 1:1 to either the test (SQ) group or placebo group (65 subjects in each group) on the enrollment day (baseline, T0), and supplemented orally with SQ or placebo products for 8 weeks. During the study period, 2 subjects in each group voluntarily withdrew consent; thus, a total of 126 (63 subjects in each group) completed the follow-up (T8). The study was in full compliance with the principles laid out in the Declaration of Helsinki and was performed at the Department of Food and Nutrition at Yonsei University (Seoul, Korea). The study protocol was approved by the Institutional Review Board (IRB) at Yonsei University (IRB approval No.: 7001988-201907-HR-631, date of approval: 4 July 2019), and registered at Clinical Research Information Service (CRIS, http://cris.nih.go.kr. Accessed date: 11 May 2021; clinical trial No.: KCT0006122). Before participation in the study, all subjects signed a written informed consent form.

### 2.2. Study Subjects and Recruitment Criteria

Participants were screened and selected based on the criteria for this study. Invitation to participate in the study was mainly based on a local advertisement. The study population included healthy adult Korean subjects without obesity aged 50 to 75 years. During the screening visit, immune status as assessed by the white blood cell (WBC) count was also considered the main criterion. According to clinical laboratory reference value reports of Korean subjects, WBC counts of 3 × 10^3^/µL to 8 × 10^3^/µL were established as ‘normal immune status’ and used as recruitment criteria [20]. Exclusion criteria were influenza-prevention injection in the 3 months before study entry, body mass index (BMI) under 18.5 kg/m^2^ or over 30 kg/m^2^ (underweight or obese), any clinically significant history or treatment of acute or chronic cardiovascular disease, metabolic disease, liver disease, kidney disease, hematological disease, respiratory disease, urinary disease, digestive disease, and oncological disease, regular consumption of food supplements for enhancing immune status in a month before inclusion in the study, antipsychotic drug treatment within 3 months before the screening test, pregnancy or breastfeeding, and known or suspected allergy to any ingredient of the tested products. Concomitantly prohibited drugs (immunosuppressants) were also set as follows: Prednisolone, Calcot, Prandin, Imuran, Neoral, Cypol N, Implanta, Prograf, Tacrobell, CellCept, Mypoltic, Bredinin, Sirolimus (Rapamune), Everolimus (Certican), Mizoribine, Anti-lymphocyte globulin (ALG), Anti-thymocyte globulin (ATG), Muromonab-CD3 (OKT3), Seamurect, and Xenafax.

### 2.3. Group Size Determination

The group size for this study was calculated by referring to our previous clinical trial [19]. To calculate the number of each group size, the change in NK cell cytotoxicity after supplementation with SQ or placebo for 8 weeks was assumed to be 11.1% and 9.39% for the SQ group and the placebo group, respectively. The standard deviation between the test group and placebo group was assumed to be 2.89% from the referred publication. A significance level (α) of 0.05 was determined using a two-sided test, type 2 error (β) was set to 0.2, and the power of the test was maintained at 80%. Consequently, the sample size per group was 45 participants. Assuming a dropout rate of 30%, the target sample size for registration is 65 per group, which is equivalent to 130 total participants.

### 2.4. Study Interventions and Materials

Through strict calculations of group size, this study confirmed the total number of 130 subjects and the 1:1 ratio randomization design. The allocation information based on a blocked randomization method was completely confidential to researchers and subjects of this study during the intervention (double-blinded), and only the study material’s manufacturer (WORLDWAY Co., Ltd.; Sejong-si, Korea) knew the allocation information and did not disclose it until the completion of the study. All subjects orally consumed 7.5 g of SQ or placebo products per day (3.75 g each before breakfast and dinner, looks no difference in appearance) for 8 weeks. A cutoff range of compliance was set to over 80%, and subjects with compliance of less than 80% were excluded from the final analysis. Additionally, subjects who were unsuitable to include for the final analysis were also excluded (Figure 1).

The main ingredient of SQ, silk peptide, is extracted and hydrolyzed from the silkworm, *Bombyx mori* L. Silk peptides are a natural complex of amino acids that are reported to be manufactured as processed foods and have been manufactured and sold for 10 years as the main ingredient in processed foods or supplementary ingredients for healthy functional foods (KMFDS manufacturing report Nos. 2002025703010 and 2002025703074). The free amino acid composition of the SQ used in this study is known as follows: glycine, 336.53 mg; alanine, 297.97 mg; serine, 157.34 mg; other free amino acid components, 28.11 mg per gram [10]. All nutrient and component information was verified by the Korea Health Supplement Institute (certified as Food Testing Laboratory by KMFDS and Korea Laboratory Accreditation Scheme). Study materials were formulated and produced by WORLDWAY Co., Ltd. (Sejong-si, Korea) following good manufacturing practice (GMP) guidelines (Authorization No. 20050021). Additionally, the manufacturing process was HALAL-certified and International Organization for Standardization—Food Safety Management System (ISO FSMS 22000)-approved. The preparation of the test product and amino acid analysis were detailed in previously published articles [9,19].

### 2.5. Safety of SQ and Dose Calculation

A summary of the experimental toxicity test is shown in Table 1. As for technical ease such as autopsy, organ observation, and histopathological examination, rats (because their size is bigger than mice) were used for the toxicity tests. No practical side effects of concern were found. To determine the proper dose for the current RCT, we used the results of in vivo and ex vivo experiments performed to confirm the efficacy of SQ supplements on immune enhancement [9]. In the study, mice were used due to their relative ease of handling compared to rats. The mice orally consumed low to high doses of SQ [0, 750, 1500, 3000, and 7500 mg SQ/kg body weight (bw)/day] for 2 months; as a result, consuming 750 and 1500 mg SQ/kg bw/day efficiently enhanced NK cell activity, with an improvement of T helper 1-type cytokines (IL-2 and IFN-γ) expression. Therefore, 1500 mg of SQ for mice was converted and confirmed to be a human daily dose of 7.5 g. Indeed, our previous human preliminary trial [19] was conducted with a daily dose of 9 g, and all anthropometric and biochemical data revealed no abnormal findings; therefore, 7.5 g/day of SQ supplementation was thought to be safe.

### 2.6. Anthropometric Parameters, Physical Activity, and Dietary Intake

All subjects were instructed to fast for 12 h before the anthropometric checkup and blood sample collection. For anthropometric parameters, body weight and fat percentage were measured by a body composition monitor scale (UM0703581; Tanita, Tokyo, Japan) with subjects wearing lightweight clothes. Height was also measured by a GL-150 stadiometer (G-tech International, Uijeongbu, Korea) while the participants were not wearing shoes. Then, BMI was calculated (kg/m^2^). For vital signs, systolic and diastolic blood pressure (BP) and pulse were measured using an automatic BP monitor (FT-200S; Jawon Medical, Gyeongsan, Korea). Body temperature was checked using a Thermofinder (FS-300; Hubidic Ltd., Anyang, Korea).

Through the Global Physical Activity Questionnaire (GPAQ), the metabolic equivalent task (MET) value was used for physical activity evaluation in units of min/week [21]. The GPAQ is a questionnaire developed to measure physical activity and is composed of questions such as leisure activities, household activities, activities related to work, and activities when moving to a place [22]. The MET value represents the relative ratio of the working metabolic rate to the resting metabolic rate of a person, and the higher the MET value is, the higher the amount of physical activity.

A dietary intake (3-day food record) survey was conducted to evaluate the food and nutrient intake status of the study subjects during participation using CAN-Pro software ver. 3.0 (The Korean Nutrition Society, Seoul, Korea).

### 2.7. Blood Collection, Hematology, Biochemical Parameters, and Urinalysis

Venous blood specimens were collected in ethylenediaminetetraacetic acid (EDTA)-coated and plain tubes and stored at −80 °C until analysis. Hematology, including WBC, lymphocyte, monocyte, granulocyte, and red blood cell (RBC) counts, hemoglobin levels, hematocrit, and platelet counts, was analyzed using an automated blood counting analyzer (HORIBA Ltd., Tokyo, Japan).

For biochemical data, alanine aminotransferase (ALT), aspartate aminotransferase (AST), and alkaline phosphatase (ALP) levels were measured using the International Federation of Clinical Chemistry and Laboratory Medicine (IFCC) UV method, with a HITACHI 7600 automatic analyzer (Hitachi Ltd., Tokyo, Japan). Total protein concentrations were measured with the Biuret method using a Cobas 8000 c702 automatic analyzer (Roche Ltd., Basel, Switzerland). Blood glucose levels were measured in venous blood samples collected from patients after a 12 h fast using the hexokinase method, with a HITACHI 7600 automatic analyzer (Hitachi Ltd., Tokyo, Japan). Total cholesterol and triglyceride levels were measured with an enzymatic method using an Auto Chemistry Analyzer Express Plus (Chiron Diagnostics Co., Walpole, MA, USA). High-density lipoprotein (HDL) cholesterol levels were measured with a homogeneous enzymatic colorimetric method using HDL-C plus Gen.3 (Roche Diagnostics Ltd., Rotkreuz, Switzerland). Blood urea nitrogen (BUN) levels were measured using a HITACHI 7600 automatic analyzer (Hitachi Ltd., Tokyo, Japan) with a kinetic UV assay for urea and urea nitrogen levels. Creatinine levels were measured by measuring the concentration of the reaction product with picric acid in an alkaline solution at a wavelength of 505 nm, with a HITACHI 7600 automatic analyzer (Hitachi Ltd., Tokyo, Japan).

The URiSCAN system (URiSCAN 10 SGL Strip; YD Diagnostics, Yong-In, Korea) was used to evaluate urine specimens for the diagnosis of renal diseases and systemic adverse effects. The urine specimens were analyzed for specific gravity and pH.

### 2.8. Primary and Secondary Endpoints

NK cell cytotoxicity, a signature measurement of immune status, was used for the primary endpoint, and effector cell (E) to target cell (T) ratios (E:T ratios) of 1.25:1, 2.5:1, 5:1, and 10:1 were selected. The basic principle and ratio of NK cell cytotoxic activity experiments followed the official guidelines published by KMFDS regarding the evaluation of NK cell cytotoxicity (Guideline for Functional Ingredient Evaluation on Immune Enhancement (Notification ID: Guide-0779-01)) and the assay kit manufacturer’s instructions regarding methodology. We used a CytoTox 96^®^ Non-Radioactive Cytotoxicity Assay (G1782; Promega Co., Madison, WI, USA), a colorimetric method, as an alternative to radioactive cytotoxicity assays. The assay quantitatively measures lactate dehydrogenase (LDH), a stable cytosolic enzyme that is released upon cell lysis, in much the same way that ^51^Cr is released in radioactive assays. Additionally, the result of the colorimetric assay was reported to be identical to the radioactive method [23,24] and requires no radioactive waste disposal. Detailed information on how isolated peripheral blood mononuclear cells (PBMCs, E) from whole blood react to and are cultivated with K562 cells (T; Korean Cell Link Bank, Seoul, Korea) in various E:T ratios of 1.25:1 to 10:1 was previously reported by our research team [25].

Interferon (IFN)-γ was measured with an IFN-γ High-Sensitivity Human Enzyme-Linked Immunosorbent Assay (ELISA) kit (Kit30173/00100540; Abcam plc, Cambridge, UK) according to the manufacturer’s instructions. Interleukin (IL)-12 levels were analyzed by a High-Sensitivity Human IL-12 (P70) ELISA kit (EK0421; Genway Biotech Inc., San Deigo, CA, USA) read at 450 nm using a Victor X5 2030 multilabel plate reader (PerkinElmer, Inc., Hopkinton, MA, USA). IL-2 levels were confirmed using a Human IL-2 ELISA Kit (Cusabio Biotech, Houston, TX, USA) and read using a Victor X5 2030 multilabel plate reader (PerkinElmer, Inc., Hopkinton, MA, USA). IL-6, IL-1β, and tumor necrosis factor (TNF)-α levels in serum were measured using a MILLIPLEX Kit (Cat#: HSTCMAG-28SK; Millipore Co., Billerica, MA, USA). Immunoglobulin (Ig) G1, IgG2, and IgM were measured by the immune nontuberculous method using a Bep II instrument (BEP 2000 Advance System; SIEMENS, Tarrytown, NY, USA).

### 2.9. Analysis Group and Statistical Method

Among the 126 subjects (63 subjects in each group) who completed this 8-week intervention study, 2 and 6 subjects in the SQ and placebo groups respectively, were excluded because they violated the study protocol (Figure 1). Thus, our results were executed by the group size of 61 (SQ group) vs. 57 (placebo group) for statistical analysis (total 118 subjects).

All data were analyzed using SAS^®^ (Version 9.4; SAS Institute, NC, USA). Chi-squared tests were used for noncontinuous variables, and the noncontinuous variables were presented as numerical values or percentages (%) for a descriptive purpose. Paired and independent *t*-tests were used for within- and between-group comparisons respectively, in continuous variables, and the continuous variables were presented as mean ± standard deviation (SD) for a descriptive purpose. To adjust baseline differences for changed values (Δ: changes from T0 at T8), analysis of covariance (ANCOVA) was used. Two-tailed *p* < 0.05 were considered to be statistically significant.

## 3. Results

The intervention was started on 2 January 2020 and finished on 16 June 2020. As described before, we screened and enrolled 137 and 130 subjects, respectively. Among the 130 subjects, 126 subjects completed their participation, and finally, 118 subjects were included for the final analysis (Figure 1).

### 3.1. Baseline Information on Subjects

The results of demographic and anthropometric information are summarized in Table 2. Of the subjects participating in this study, 7 were male (2 in the SQ group, 5 in the placebo group), and 111 were female (59 in the SQ group and 58 in the placebo group). The total subjects’ mean age was 56.45 years old, and there were no significant differences between the SQ and placebo groups. Verifying the results of demographic and anthropometric data, it was judged that the selection and allocation of subjects were appropriately made, and there was no significant difference between the two groups in any baseline values.

### 3.2. Dietary Intake and Physical Activity

Table 3 outlines the results of the dietary intake survey and physical activity questionnaire at T0 and T8. Since metabolic and hormonal alterations can influence clinical results, a dietary- or exercise-related survey was conducted and analyzed. Δ Dietary intake (calories, carbohydrate, protein, lipid, and fiber) and Δ MET (physical activity) did not significantly differ between the groups. Regarding hormonal alterations, 49 and 41 subjects in the SQ and placebo groups respectively, were menopausal. Only 1 subject in the placebo group was receiving hormone treatment during the intervention period (data not shown).

### 3.3. Sil-Q1 Efficacy Test: NK Cell Cytotoxicity and Cytokines

NK cell cytotoxicity, the primary efficacy endpoint, and other secondary endpoints were measured at T0 and T8, and the analysis results are shown in Table 4. The NK cell cytotoxicities at all E:T ratios in the SQ group were significantly increased at T8 compared to those at T0 (E:T = 10:1, *p* = 0.002; E:T = 5:1, *p* = 0.004; E:T = 2.5:1, *p* = 0.023; E:T = 1.25:1, *p* = 0.030), whereas no significant changes were observed in the placebo group during the study period. Furthermore, the Δ NK cell cytotoxicities at E:T ratios of 10:1, 5:1, and 2.5:1 in the SQ group were greater than those in the placebo group (*p* = 0.022, 0.001, and 0.016, respectively).

Regarding a cytokine variable, the IL-12 level in the SQ group was significantly increased after the intervention (*p* = 0.041). Additionally, Δ IL-12 in the SQ group was significantly greater than the placebo group (*p* = 0.023) (Table 4). Other parameters (TNF-α, IL-1β, IL-6, IL-2, IFN-γ, IgG1, and IgG2) which did not show significant differences in changed values (Δ) between the two groups are shown in the supporting data (Supplementary Table S1).

## 4. Discussion

The novel findings of this confirmatory RCT indicated that daily supplementation with 7.5 g of Sil-Q1 enhanced NK cell cytotoxicity and IL-12 levels. These findings are in line with our notion and study hypothesis that SQ supplementation will improve the functions of NK cells and stimulate cytokines related to T cells.

Providing amino acids through nutritional supplements has received considerable interest from the elderly population and has been widely applied [26]. Few articles have elucidated the efficacy of amino acid supplementation and immuno-stimulation, such as isolated amino acids [27,28] and a few amino acid combinations [29,30]. Although the efficacy of certain amino acids in increasing immunocompetence was clearly demonstrated by some studies, others still show inconclusive results [31]. Since amino acids are utilized in key metabolic pathways of the immune system, certain amino acid imbalances or antagonistic effects can have a negative impact. Thus, supplementing amino acids with functional foods derived from nature rather than isolated single amino acids may be a safer application, but this also requires special attention through elaborate dose determination, sophisticated safety analysis, and scientific efficacy verification.

A confirmatory RCT is considered the cornerstone of scientific human research, can contribute to evidence-based medicine (EBM), and strongly influences numerous clinical indications/guidelines [32]. Since the human body does not always respond consistently to a given intervention, this unpredictability/heterogeneous matter of clinical trials challenges clinical researchers. Nevertheless, RCTs remain a straightforward solution and gold standard [33]. The basic principles of strong RCTs are associated with the comparison of two or more regimens (active vs. traditional treatment/placebo) under controlled conditions, randomization and blinding, and statistical analysis [34].

Unlike drug development research, which is divided into several phases [35], repeated clinical trials of functional foods are seldom carried out and rarely found. Thus, considering the constraints of cost, time, ethical requirements, and limits to the duration of treatment and follow-up, our confirmatory RCT with SQ is a very exceptional and profound study. Through the current confirmatory RCT, we had the opportunity to discuss the results in a comprehensive view.

WBCs and their subpopulations are regarded as a representative component of immune markers [36] and are also associated with vulnerability and mortality in elderly adults [37,38,39]. Thus, this study included subjects with a WBC range of 3 × 10^3^/µL to 8 × 10^3^/µL and age over 50 years old as the selection criteria. Due to the limitations of local advertising and the time to participate in the study (daytime on weekdays), mainly middle-aged women were enrolled.

The most important part of the results is that the primary endpoint (NK cell cytotoxicity) outcome was more definitively validated in confirmatory RCTs with a large group size. In our previous human RCT, sub-analysis with non-influenza-vaccinated subjects showed the immunomodulatory effect of SQ [19]. NK cell cytotoxicity, which promotes cytotoxicity and represents a crucial element of innate immunity [40], is a very sensitive and accurate indicator of immune status in the elderly [41]. The data previously showed that the E:T ratio of 10:1 in NK cell cytotoxicity was significantly increased within the test group (30.7 ± 2.28 to 41.8 ± 2.71), and the cytotoxicity of the test group showed a greater increase in the change value than that of the placebo group (2.65 ± 2.71 vs. test group, 11.1 ± 2.89) after 8 weeks of supplementation. Furthermore, our current results showed a greater increase in NK cell cytotoxicity at three E:T ratios of 10:1, 5:1, and 2.5:1. There was a concern that the baseline NK cell cytotoxicities in the placebo group seemed higher than those in the SQ group, so that these values hid the enhancement of cytotoxicity in the placebo group. However, no statistically significant changes were observed in the placebo group during the intervention period, and even though the baseline NK cell cytotoxicity at an E:T ratio of 5:1 in the placebo group was significantly higher than that in the SQ group, an increase in NK cell cytotoxicity at this E:T ratio in the SQ group was still greater than the placebo group after the baseline adjustment (Table 4). Therefore, considering the two conclusive outcomes of the increase in NK cell cytotoxicity, it can be said that SQ contributes greatly to the immunity enhancement of elderly participants.

In specific conditions of age (i.e., infancy or elderly), the presence of environmental stimuli (i.e., exercise and physical stress) [42], or in illness status [43], nonessential amino acids can be a conditional or semi-essential nutrient. Most components of SQ are mainly free amino acids, where glycine, alanine, and serine are the main components.

Glycine, which accounts for over 30% of SQ, itself is a potent antioxidant [44] and synthesizes antioxidative molecules, such as glutathione. In particular, the glycine-participating chloride channel in leukocytes regulates cytokine production and the overall immune functions of leukocytes [45]. Though clinical trials are lacking, in vitro studies have shown that the extracellular glycine concentration activated and hyperpolarized the plasma membrane of macrophages, monocytes, lymphocytes, and neutrophils [46], and that glycine-concentrated culture medium enhances B cell antibody production [47]. Additionally, in vivo experiments with dietary supplementation with 1% [48] or 5% glycine [49] in rats reported reduced inflammation and an improved survival rate. These findings suggest that glycine supplementation may provide free radical scavenging activity and antioxidative defense power for leukocytes.

Alanine is an essential precursor for synthesizing glucose in hepatocytes [50]. Sufficient hepatic glucose becomes an energy substrate for immune-related cells, such as leukocytes [51]; thus, alanine influences the overall immune system. In particular, although the exact mechanism has not been confirmed, alanine may have alanine-mediated actions in the inhibition of protein degradation in immunocytes [52,53]. However, the results of oral alanine supplementation are rarely reported. Although some studies described immune-enhanced outcomes after an alanine-containing total parenteral nutrition application, it was limited to combined treatment with L-alanyl-L-glutamine dipeptide in critically ill patients [54,55]. These results suggest that the successful use of alanine is effective in improving health and preventing infectious diseases.

Serine, especially phosphatidylserine, plays a structural role in biological membranes and is known to act as a cofactor for signaling enzymes essential for various cellular functions [56]. Additionally, phosphatidylserine is utilized in activating T cells and increasing antibody production. Collectively, though essential amino acids are found in small amounts, the experimental findings of the major amino acid components of SQ, glycine, alanine, and serine, support the mechanism of improved immunity.

NK cells, by themselves, contain perforins and granzymes, and the cytotoxic properties of these contents induce cell death [57] and synergistically trigger endogenous programmed cell death [58]. Additionally, NK cells participate as an essential factor in the prevention of tumor initiation and evolution by killing (lytic activity, apoptosis, and exocytosis) and cytokine secretion. Recently, the role of NK cells in upholding the T helper type 1 cell subtype [59,60] and the ability to recognize antibody-bound antigens have become interesting research points [61].

In our current study, together with NK cell activation, IL-12 levels were elevated after SQ supplementation and showed a significant difference between the SQ group and the placebo group. In addition, in our previous RCT, IL-12 was also increased after SQ supplementation [19]. IL-12 is an inflammatory cytokine mainly produced by antigen-presenting cells (APCs; macrophages, dendritic cells, and B cells). IL-12 mediates the development of T helper type 1 (Th1) cells, which preferentially secrete IFN-γ and IL-2, and Th1 cells are involved in the activation of macrophages, one of the APCs. Meanwhile, both IL-2 and IL-12 have been reported to stimulate cytotoxicity activity of NK cells that produce proinflammatory cytokines, such as TNF-α and IFN-γ. However, our study only showed an increase in IL-12 levels with NK cell cytotoxicity enhancement. Unfortunately, we could not clearly elucidate where IL-12 exactly releases from and why it was particularly increased in the present study because we did not observe changes in APCs, the source of IL-12. Additionally, cytokine production of T cells has a wide range [62], and it is difficult to identify cytokine values in whole-blood measurements rather than cytokine analysis after isolated PBMC stimulation [63]. Thus, further study is needed to find an exact mechanism for NK cell activation and cytokines after SQ supplementation.

Moreover, antibody-dependent cell-mediated cytotoxicity is also noted as an important immune function of NK cells, and some reports elucidated B cell- and antigen-mediated immune enhancement after amino acid treatment [64,65,66]. However, there was no relationship between increases in NK cell cytotoxicity and immunoglobulin alterations. The inclusion of relatively healthy people who were not in illness status [67] and the nature of immunoglobulins to maintain homeostasis [68] might have made it difficult to observe the change. Given that NK cells sit at the crossroads of innate and adaptive immune responses and that NK cell cytotoxicity greatly increased in our study, the immune-enhancing effect of SQ can be said to be promising.

The limitations and future applications included in our RCTs are as follows. To capture multifaceted improvements in immune parameters after SQ supplementation and to verify the effects of SQ supplementation in the real world, subjects with various health conditions, a proportional sex distribution, stratified analysis according to sex, and more age groups are needed, with multicenter involvement. Additionally, since an advanced cytokine experiment could not be conducted in this study because of the limited blood volume, a detailed analysis of cytokine production is still warranted to explore cytokine-related mechanisms of SQ supplementation. Furthermore, to see acute immunoglobulin changes, if safe human clinical trials are possible, we can also suggest confirming the response after a single administration of multiple doses. Finally, trained interviewers guided the study subjects to maintain their daily life as usual during the intervention period; however, MET values seemed to have somewhat high disparities, although there was no statistical significance. The MET values’ disparities might be able to affect the immune response; thus, a more controlled design for physical activity should be considered.

In conclusion, our confirmatory RCT with controlled interference factors and reduced dose showed substantial practical implications of Sil-Q1 supplementation. In conclusion, Sil-Q1 is an effective and safe functional food supplement for enhancing immune function.

## Figures and Tables

**Table 1:** Toxicity test results of Sil-Q1.

Type of Test		Species	Dose	Test Result
Single administration	Rodent	SD Rat (Male (M) 15, Female (F) 15)	0, 2000, 5000 mg/kg/body weight (bw)	No deaths, general symptoms, weight change, and autopsy findings Approximate lethal dose (ALD) > 5000 mg/kg bw/day
Dose-range finding (4 weeks)	Rodent	SD Rat (M 10, F 10)	0, 500, 1000, 2000 mg/kg/bw	No deaths, weight, feed intake, water intake, ophthalmic examination, and autopsy findings No observed adverse effect level (NOAEL) > 2000 mg/kg bw/day
Repeated administration (13 weeks)	Rodent	SD Rat (M 40, F 40)	0, 500, 1000, 2000 mg/kg/bw	No body weight, feed intake, urinalysis, ophthalmological test, hematological test, blood biochemical test, blood coagulation time test, organ weight, histopathologic test, and autopsy findings NOAEL > 2000 mg/kg bw
Genotoxicity	Ames test	*S. Typhimurium* TA98, TA100, TA1535, TA1537 *E.coli* WP2 uvrA	0~5000 μg/plate	Does not cause reverting mutations
	Chromosomal abnormality test	CHL cells	0~5000 μg/mL	Does not cause chromosomal abnormalities on CHL cells
	Micronucleus test	ICR mouse bone marrow cells	1250, 2500, 5000 mg/kg	Does not induce micronuclei in bone marrow cells of ICR mice

**Table 2:** Baseline information on subjects.

	SQ Group (*n* = 61)	Placebo Group (*n* = 57)	Total (*n* = 118)	*p*-Value *^a^*
Male/Female (*n*, %)	2 (3.28)/59 (96.72)	5 (8.77)/52 (91.23)	7 (5.93)/111 (94.07)	0.261 *^b^*
Age (years)	55.90 ± 4.68	57.04 ± 4.65	56.45 ± 4.68	0.190
Height (cm)	157.51 ± 5.41	158.07 ± 5.53	157.78 ± 5.45	0.578
Weight (kg)	58.03 ± 7.26	59.85 ± 8.27	58.91 ± 7.78	0.207
BMI (kg/m^2^)	23.36 ± 2.47	23.85 ± 2.57	23.60 ± 2.52	0.294
Waist circumference (cm)	85.98 ± 6.55	87.60 ± 6.80	86.76 ± 6.69	0.191
Hip circumference (cm)	95.07 ± 4.33	96.31 ± 5.02	95.67 ± 4.70	0.153
Fat mass (%)	32.38 ± 4.53	33.04 ± 4.60	32.69 ± 4.56	0.433
Currently drinking (*n*, %)	6 (9.84)	7 (12.28)	13 (11.02)	0.672 *^c^*
Currently smoking (*n*, %)	0 (0.00)	2 (3.51)	2 (1.69)	0.231 *^b^*
Prescription compliance (%)	96.05 ± 4.55	94.50 ± 5.43	95.30 ± 5.03	0.095

Values are presented as the mean ± standard deviation (SD) or number (%). *^a^* Analyzed by an independent *t*-test between the groups. *^b^* Analyzed by Fisher’s exact test between the groups. *^c^* Analyzed by a chi-square test between the groups. BMI: body mass index. SQ: Sil-Q1.

**Table 3:** Dietary intake and physical activity changes.

	SQ Group (*n* = 61)			Placebo Group (*n* = 57)			*p*-Value *^b^*
	T0	T8	*p*-Value *^a^*	T0	T8	*p*-Value *^a^*	
Dietary intake							
Calorie (kcal)	1871.49 ± 114.89	1854.92 ± 117.46	0.081	1889.91 ± 121.31	1852.97 ± 105.72	0.001	
∆	−16.58 ± 72.82			−36.94 ± 75.12			0.138
Carbohydrate (g)	287.50 ± 18.41	285.31 ± 18.05	0.173	291.06 ± 19.12	284.84 ± 16.01	0.001	
∆	−2.20 ± 12.46			−6.23 ± 12.30			0.080
Protein (g)	73.72 ± 4.77	73.78 ± 4.92	0.903	74.56 ± 5.03	73.38 ± 4.52	0.033	
∆	0.06 ± 3.86			−1.18 ± 4.08			0.091
Lipid (g)	47.09 ± 3.53	46.34 ± 3.84	0.083	47.18 ± 3.88	46.44 ± 3.60	0.155	
∆	−0.75 ± 3.33			−0.74 ± 3.88			0.988
Fiber (g)	11.18 ± 1.94	11.36 ± 2.21	0.605	11.49 ± 1.96	11.29 ± 2.25	0.614	
∆	0.17 ± 2.57			−0.19 ± 2.90			0.469
Physical activity							
MET (min/week)	2942.95 ± 3536.50	3097.70 ± 3583.62	0.623	3370.88 ± 4008.54	3943.86 ± 5940.32	0.497	
∆	154.75 ± 2443.23			572.98 ± 6327.03			0.642

Values are presented as the mean ± standard deviation (SD). *^a^* Analyzed by a paired *t*-test between T0 and T8 within each group. *^b^* Analyzed by an independent *t*-test for change value between the groups. Δ (delta) represents changed values (the change from T0 to T8). MET: metabolic equivalent task. SQ: Sil-Q1. T0: baseline. T8: follow-up.

**Table 4:** Efficacy results of NK cell cytotoxicity and cytokine (IL-12).

	SQ Group (*n* = 61)			Placebo Group (*n* = 57)			*p*-Value *^b^*
	T0	T8	*p*-Value *^a^*	T0	T8	*p*-Value *^a^*	
NK cell cytotoxicity E:T ratio (%)							
10:1	41.83 ± 12.75	49.60 ± 18.23	0.002	45.87 ± 17.57	45.24 ± 17.41	0.817	
∆	7.77 ± 18.83			–0.63 ± 20.52			0.022
5:1	27.43 ± 9.60	32.41 ± 12.08	0.004	32.28 ± 14.82 *	28.87 ± 11.14	0.144	
∆	4.98 ± 12.88			–3.41 ± 17.33			0.001 *^c^*
2.5:1	17.85 ± 7.11	20.65 ± 7.58	0.023	20.79 ± 10.87	18.55 ± 7.29	0.188	
∆	2.81 ± 9.39			–2.24 ± 12.70			0.016
1.25:1	11.36 ± 5.35	13.41 ± 5.96	0.030	13.01 ± 7.44	12.37 ± 5.42	0.587	
∆	2.05 ± 7.21			–0.64 ± 8.80			0.071
Cytokine							
IL-12 (pg/mL)	12.13 ± 18.31	20.82 ± 40.97	0.041	13.69 ± 33.03	11.49 ± 27.95	0.329	
∆	8.70 ± 32.44			–2.20 ± 16.89			0.023

Values are presented as the mean ± standard deviation (SD). *^a^* Analyzed by a paired *t*-test between T0 and T8 within each group. *^b^* Analyzed by an independent *t*-test for change value between the groups. *^c^* Analyzed by ANCOVA (adjusted on baseline NK cell activity 5:1) between the groups. * Statistically different at baseline (*p* = 0.039), and the other E:T ratios of NK cell cytotoxicity showed nonsignificant differences at T0. Δ (delta) represents changed values (the change from T0 to T8). IL: interleukin. NK cell: natural killer cell. SQ: Sil-Q1. T0: baseline. T8: follow-up.

## Data Availability

The data presented in this study are available on request from the corresponding author.

## Supplementary Materials

The following are available online at https://www.mdpi.com/article/10.3390/nu13092930/s1, Table S1: Results of other cytokines and immunoglobulins (secondary endpoints).
