# Supplementary material for: Supplementation with a Natural Source of Amino Acids, Sil-Q1 (Silk Peptide), Enhances Natural Killer Cell Activity: A Redesigned Clinical Trial with a Reduced Supplementation Dose and Minimized Seasonal Effects in a Larger Population
Source: Nutrients. 2021 Aug 24;13(9):2930. doi: 10.3390/nu13092930 (PMC8466343; doi:10.3390/nu13092930)
Supplement: Supplementary file 1 [file nutrients-13-02930-s001.zip › nutrients-1265681-supplementary.pdf]

Supplementation with a natural source of amino acids, Sil-Q1 (silk peptide), enhances natural killer cell activity: a redesigned clinical trial with a reduced supplementation dose and minimized seasonal effects in a larger population

Jung Min Cho[1,†], Dokyeong Yoo[1,†], Jeong-Yong Lee[2], Mi-Sun Oh[2], Ki-Chan Ha[3], Hyang-Im Baek[3], Seung-Min Lee[4,5], Jong Ho Lee[1,5], Hye Jin Yoo[1,5,*]

Online Supplementary Material

Supplementary Table S1. Results of other cytokines and immunoglobulins (secondary endpoints)

	SQ group (*n*=61)				Placebo (*n*=57)				*p*-value[b]
	T0	T8	Δ	*p*-value[a]	T0	T8	Δ	*p*-value[a]	
IL-2 (pg/mL)	31.35 ± 19.50	33.90 ± 18.75	2.56 ± 10.45	0.061	34.16 ± 16.66	39.58 ± 20.58	5.42 ± 10.38	0.001	0.139
IFN-γ (pg/mL)	5.97 ± 6.36	4.20 ± 3.31	-1.76 ± 4.36	0.003	6.38 ± 6.70	5.54 ± 5.98	-0.84 ± 6.96	0.368	0.887
TNF-α (pg/mL)	0.63 ± 0.17	0.77 ± 0.40	0.13 ± 0.40	0.012	0.68 ± 0.22	0.80 ± 0.45	0.12 ± 0.47	0.055	0.393
IL-6 (pg/mL)	1.68 ± 2.13	1.41 ± 1.02	-0.27 ± 2.09	0.314	1.49 ± 0.79	1.45 ± 0.80	-0.04 ± 0.56	0.606	0.405
IL-1β (pg/mL)	0.06 ± 0.03	0.05 ± 0.05	0.00 ± 0.06	0.564	0.07 ± 0.06	0.05 ± 0.04	-0.02 ± 0.07	0.028	0.185
IgG1 (mg/L)	7186.89 ± 1241.88	7333.11 ± 1278.46	146.23 ± 464.79	0.017	7630.88 ± 1436.76	7701.40 ± 1421.29	70.53 ± 496.52	0.288	0.394
IgG2 (mg/L)	4401.31 ± 1465.18	4449.34 ± 1494.48	48.03 ± 400.59	0.353	4083.68 ± 1574.20	4126.67 ± 1572.67	42.98 ± 301.73	0.287	0.938

Values are presented as the mean ± standard deviation (SD). [a] Analyzed by a paired *t*-test between T0 and T8 within each group. [b] Analysis by an independent *t*-test for change value between the groups. Δ (delta) represents changed values (the change from T0 at T8). IFN: interferon. Ig: immunoglobulin. IL: interleukin. SQ: Sil-Q1. TNF: tumor necrosis factor. T0: baseline. T8: follow-up.
